# *Caenorhabditis elegans* as a model system to study post-translational modifications of human transthyretin

**DOI:** 10.1038/srep37346

**Published:** 2016-11-21

**Authors:** Andrea Henze, Thomas Homann, Isabelle Rohn, Michael Aschner, Christopher D. Link, Burkhard Kleuser, Florian J. Schweigert, Tanja Schwerdtle, Julia Bornhorst

**Affiliations:** 1 Department of Physiology and Pathophysiology, Institute of Nutritional Science, University of Potsdam, Arthur-Scheunert-Allee 114-116, 14558 Nuthetal, Germany; 2 Department of Toxicology, Institute of Nutritional Science, University of Potsdam, Arthur-Scheunert-Allee 114-116, 14558 Nuthetal, Germany; 3 Department of Food Chemistry, Institute of Nutritional Science, University of Potsdam, Arthur-Scheunert-Allee 114-116, 14558 Nuthetal, Germany; 4 Department of Molecular Pharmacology, Neuroscience, and Pediatrics, Albert Einstein College of Medicine, 1300 Morris Park Avenue, 10461 Bronx, NY, USA; 5 Institute for Behavioral Genetics, University of Colorado at Boulder, 1480 30th St., 80303 Boulder, CO, USA

## Abstract

The visceral protein transthyretin (TTR) is frequently affected by oxidative post-translational protein modifications (PTPMs) in various diseases. Thus, better insight into structure-function relationships due to oxidative PTPMs of TTR should contribute to the understanding of pathophysiologic mechanisms. While the *in vivo* analysis of TTR in mammalian models is complex, time- and resource-consuming, transgenic *Caenorhabditis elegans* expressing hTTR provide an optimal model for the *in vivo* identification and characterization of drug-mediated oxidative PTPMs of hTTR by means of matrix assisted laser desorption/ionization – time of flight – mass spectrometry (MALDI-TOF-MS). Herein, we demonstrated that hTTR is expressed in all developmental stages of *Caenorhabditis elegans,* enabling the analysis of hTTR metabolism during the whole life-cycle. The suitability of the applied model was verified by exposing worms to D-penicillamine and menadione. Both drugs induced substantial changes in the oxidative PTPM pattern of hTTR. Additionally, for the first time a covalent binding of both drugs with hTTR was identified and verified by molecular modelling.

Since a suitable post-translational modification of the polypeptide chain (post-translational protein modification, PTPM) is essential for normal folding, targeting and specificity of proteins, a misregulation of the process might have far-reaching consequences, including effects on cell growth, division and survival. Accordingly, intense focus has been directed at PTPMs, including conditions and drugs that lead to their modifications. Greater than 200 different types of PTPMs are known, ranging from enzyme-catalyzed PTPMs including phosphorylation, acetylation and glycosylation to non-enzymatic PTPMs such as oxidation, glycation, deamidation and truncation^1^. Thereby, oxidative PTPMs, in particular those with cysteine residues, are characterized as bivalent. On the one hand oxidative modifications widely regulate cellular homeostasis, yet, oxidative stress resulting in the oxidation of biomolecules along with the disruption of their biological functions might be associated with the development of diseases, such as cancer, diabetes and neurodegenerative diseases^2^,^3^,^4^. The growing interest in cysteine modifications stem from its impact on biological systems and cellular processes, including cellular signaling, proliferation, differentiation and apoptosis (summarized in ref. 5).

The patterns of cysteine modifications can be classified into reversible and irreversible oxidative PTPMs. Reversible modifications, including the formation of sulfenic acid, sulfenylamides, disulfide bridges, glutathionylated as well as nitrosylated adducts^6^, are involved in protein protection and the redox signaling cascades^7^,^8^. Additionally, there is evidence to suggest that reversible oxidative PTPMs are involved in the adaptive regulation of protein function^9^,^10^. In contrast, irreversible cysteine modifications, such as formation of sulfinic and sulfonic acid as well as adducts with tryptophane or tyrosine^6^ result in misfolding, loss of protein function, accumulation or degradation^11^,^12^, and are associated with several severe diseases including cystic fibrosis, Alzheimer’s disease, Parkinson’s disease, as well as amyotrophic lateral sclerosis^13^.

A protein that is greatly affected by oxidative PTPMs is human transthyretin (hTTR). hTTR is a visceral protein, which facilitates the transport of thyroid hormones and vitamin A in blood and cerebrospinal fluid^14^. It is a 55 kDa homotetrameric quaternary structure protein with an approximate molecular weight of 14 kDa per monomer. Each hTTR subunit reveals a free cysteine residue at position 10 (Cys10), which is susceptible to PTPMs by formation of mixed disulfides^15^. The tetramer stability depends on the kind and degree of the PTPMs at Cys10^16^, and they are recognized as potential biomarkers of several physiological and pathological aspects, such as cancer^17^, follicle and oocyte maturation^18^ and inflammation^19^. Additionally, oxidative PTPMs of hTTR have been identified as potent triggers in the formation of hTTR-related amyloid fibrils such as those observed in senile systemic amyloidosis^16^,^20^.

Nevertheless, studies on oxidative PTPMs of hTTR *in vivo* are challenging and time consuming. Therefore, herein we tested the suitability of *Caenorhabditis elegans (C. elegans*) overexpressing hTTR for this purpose.

*C. elegans* is an attractive model organism for experimental research as it may be used to study and model most human diseases (including diabetes, cardiovascular diseases, atherosclerosis, cancer and neurodegeneration) at either the metabolic or genomic level *in vivo*. It is less complex compared to the mammalian system, while 60–80% of human disease genes have homologous genes in *C. elegans*^21^. Additional characteristics that have contributed to its utility as a model include the ease of its genetic manipulability and maintenance, as well as its small size^22^. Furthermore, due to its rapid life-cycle (about 3 days) and the ability to produce 300 offspring, high-throughput analyses is feasible^23^. The aforementioned characteristics render a robust experimental model to investigate several pathways including oxidative stress, which is known to be a major contributor to several chronic human disorders^24^,^25^. Since pathways relevant to oxidative stress and oxidative stress response are highly evolutionarily conserved in the nematode, the model has contributed greatly to the understanding of drug-mediated induction of oxidative stress and oxidative stress response as well as to the identification of upstream markers of oxidative stress within interconnected signaling pathways^22^,^24^. Given that less is known about PTPMs of proteins conserved in *C. elegans*^26^,^27^,^28^, this study was designed to show drug-mediated structural changes due to PTPMs of the redox-sensitive protein hTTR. Therefore the transgenic strain CL2008 expressing high levels of hTTR was incubated with menadione (MND) or D-penicillamine and the type as well as degree of the PTPMs were studied by immunoprecipitation and matrix assisted laser desorption/ionization – time of flight – mass spectrometric (MALDI-TOF-MS) analysis of hTTR.

## Results

### Expression of hTTR in the trangenic *C. elegans* strain CL2008 and verification at the protein level

**Figure 1:**
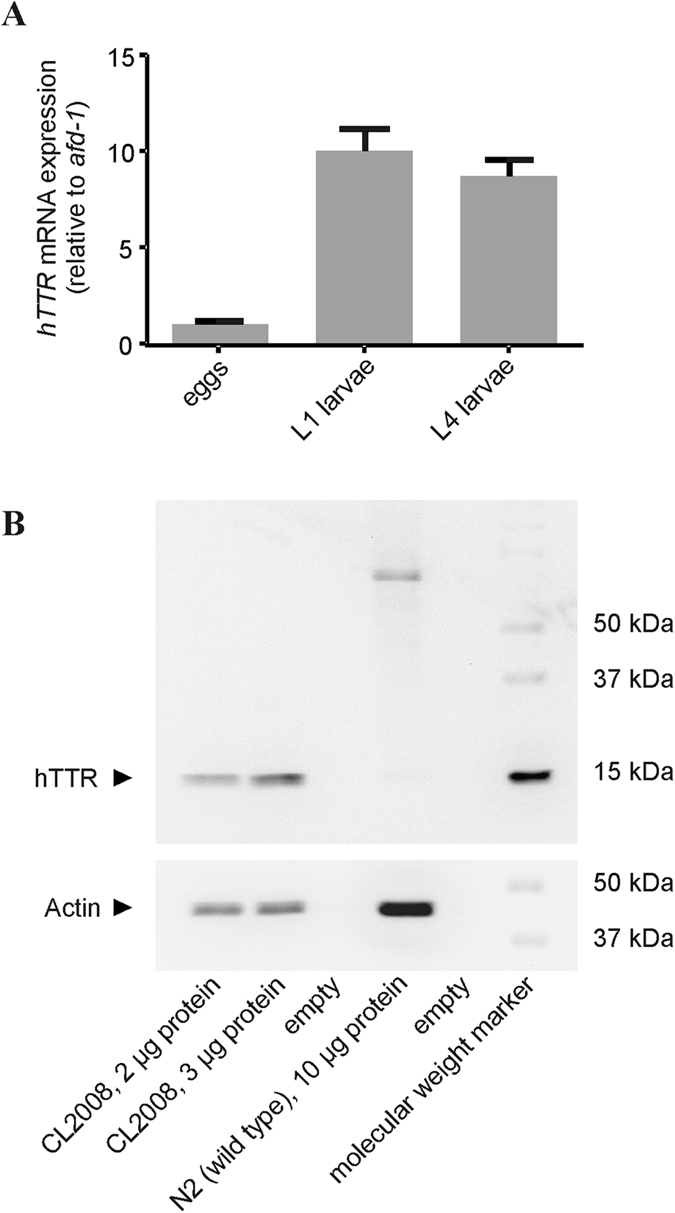
Validation of hTTR expression on mRNA (A) and protein level (B). (**A**) Relative gene expression of hTTR determined by real time RT-PCR in eggs, L1 stage and L4 stage CL2008 larvae. Shown are mean values + SD of three independent experiments in triplicate normalized relative to afd-1/β-actin mRNA. (**B**) Representative immuno blot of the transgenic *C. elegans* strain (CL2008) and the wildtype *C. elegans* strain (N2). Homogenates of CL2008 and N2 were diluted with sample buffer, sample volumes corresponding to the indicated protein concentrations were applied to and separated by SDS-PAGE. hTTR and actin were detected by immunoblotting using a polyclonal antibody specific for hTTR and a monoclonal antibody specific for actin, respectively.

The transgenic strain CL2008 with integrated copies of the unc-54/hTTR and rol-6 transgenes^29^ was analysed for its larval stage specific hTTR gene expression. Using real time RT-PCR, we showed that hTTR is predominantly expressed in the embryonic stage with a statistically indistinguishable hTTR expression in L1 and L4 stage nematodes (Fig. 1A). Additionally, the presence of hTTR in the *C. elegans* strain CL2008 has also been confirmed at the protein level as shown in Fig. 1B. In this context, the immunoblot analysis also highlights that the applied 2 and 3 μg total protein of the CL2008 extracts, respectively, is readily enough to detect hTTR. Additionally, in accordance to the mRNA analyses no hTTR or hTTR-like protein was detectable in wildtype *C. elegans* (N2 strain) even if 10 μg total protein of the N2 extracts were applied to ensure that a negative result concerning hTTR is not due to insufficient protein concentrations.

### Effect of D-penicillamine and menadione (MND) on the survival of *C. elegans* strain CL2008

**Figure 2:**
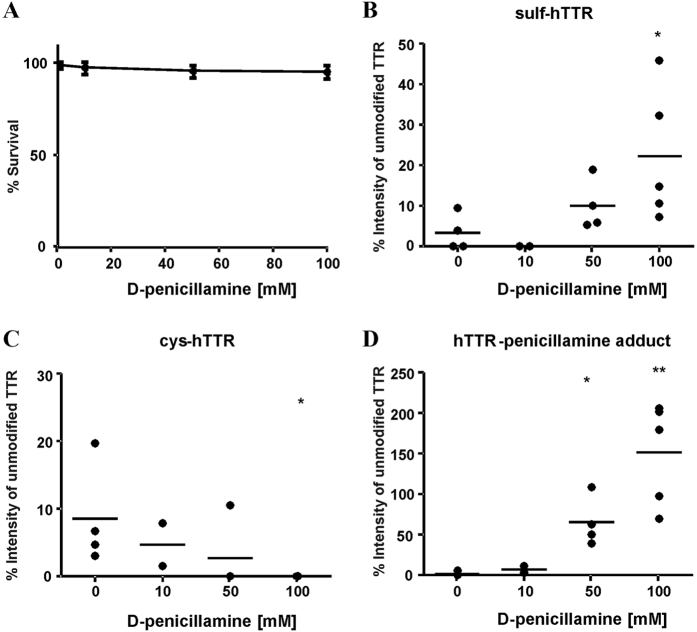
Concentration-dependent effects of D-penicillamine on lethality and post-translational modification patterns of hTTR in CL2008. (**A**) Dose–response survival curves following acute D-penicillamine exposure (1 h). All values were compared to non-treated worms set to 100% survival. Data are expressed as means ± SD from at least four independent experiments. (**B–D**) Relative amounts (intensity of the unmodified hTTR [%]) of hTTR post-translationally modified at Cys10 in homogenates of CL2008 worms following 1 h D-penicillamine incubation at L4 stage. Shown are mean values of at least three experiments each as an aligned dot blot (showing data points and the mean in form of a line). (**B**) Concentration-dependent effect of D-penicillamine on the relative intensity of sulf-hTTR. (**C**) Concentration-dependent effect of D-penicillamine on the relative intensity of cys-hTTR. (**D**) Concentration-dependent effect of D-penicillamine on the relative intensity of hTTR-penicillamine adducts. **p < 0.01, *p < 0.05 versus non-incubated CL2008 worms.

**Figure 3:**
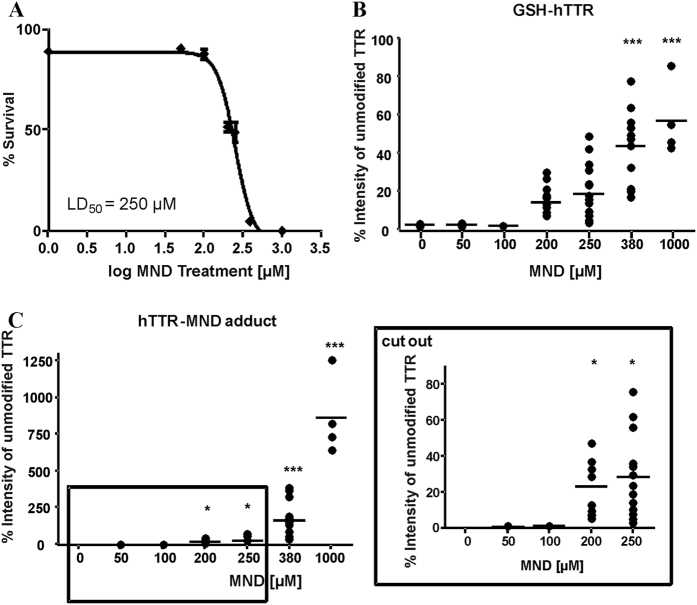
Concentration-dependent effects of MND on the lethality and post-translational modifications of hTTR in CL2008. (**A**) Dose–response survival curves and the respective LD_50_ dose following acute MND exposure (1 h). All values were compared to non-treated worms set to 100% survival and plotted against the logarithmic scale of the used MND concentrations. Data are expressed as means ± SD from at least four independent experiments. (**B,C**) Relative amounts (intensity of the unmodified hTTR [%]) of hTTR post-translationally modified at Cys10 in homogenates of CL2008 worms following 1 h MND incubation at L4 stage. Shown are mean values of at least four experiments each as an aligned dot blot (showing data points and the mean in form of a line). (**B**) Concentration-dependent effects of MND on the relative intensity of GSH-hTTR in CL2008. (**C**) Concentration-dependent effects of MND on the relative intensity of hTTR-MND adduct in CL2008. ***p < 0.001, *p < 0.05 versus non-incubated CL2008 worms.

To determine appropriate D-penicillamine and MND concentrations for dosing in the PTPM studies, CL2008 worms were treated with the respective drug for 1 h. Assessment of dose-response survival curves revealed no lethality with D-penicillamine treatments in the tested incubation range up to 100 mM (Fig. 2A). However, as shown in Fig. 3A, MND induced lethality following 1 h exposure and the dose–response survival curves exhibited an LD_50_ of 250 μM.

### Incubation with D-penicillamine and MND affects post-translational modification patterns of hTTR in CL2008

**Figure 4:**
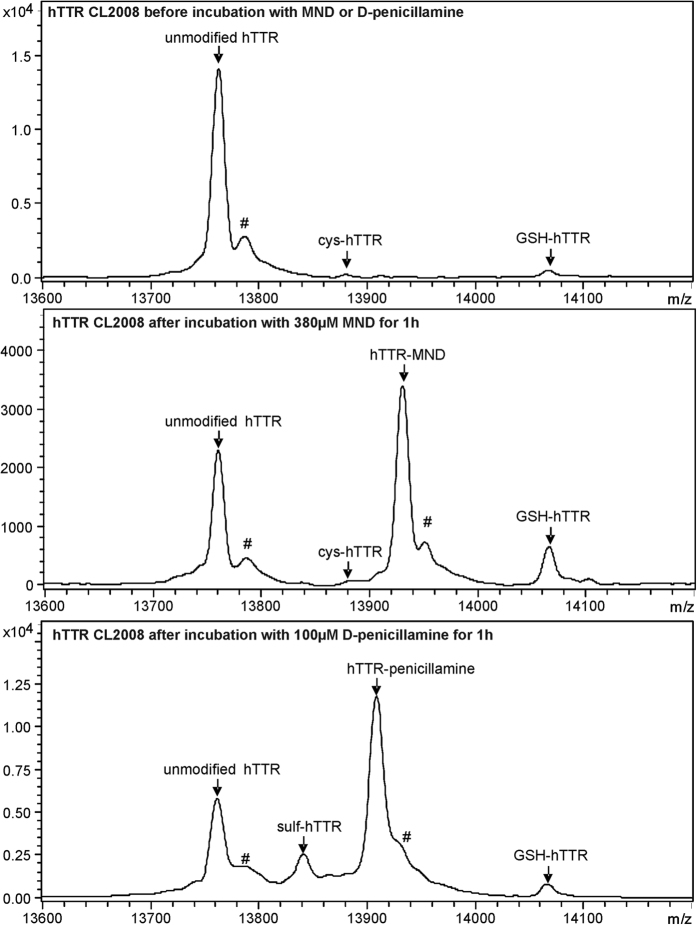
Representative MALDI-TOF mass spectra of hTTR isolated from *C. elegans* CL2008 before and after incubation with MND and D-penicillamine, respectively. According to the molecular weight and mass shift the hTTR variants were assigned as follows: unmodified hTTR (m/z 13 761.9 ± 1.4 Da); sulf-hTTR, hTTR conjugated with sulfonic acid (m/z 13 839.8 ± 2.5 Da, Δmass +78 Da); cys-hTTR, hTTR conjugated with cysteine (m/z 13 880.1 ± 2.8 Da, Δmass +118 Da); hTTR-penicillamine, adduct of hTTR and D-penicillamine (m/z 13909.4 ± 0.6 Da, Δmass +148 Da); hTTR-MND, adduct of hTTR and MND (m/z 13932.7 ± 3.0 Da, Δmass +171 Da); GSH-hTTR, hTTR conjugated with glutathione (m/z 14066.7 ± 2.3 Da, Δmass +305 Da). Sodium adducts are indicated by #.

Representative MALDI-TOF mass spectra of hTTR isolated from CL2008 are shown in Fig. 4. Besides unmodified hTTR with an average molecular weight of 13761.9 ± 1.4 Da several post-translationally modified hTTR variants and adducts could be detected. The hTTR variants were assigned according to their molecular weight and the mass difference to unmodified hTTR as follows: S-sulfonated hTTR (sulf-hTTR, m/z 13839.8 ± 2.5 Da, Δmass +78 Da), S-cysteinylated hTTR (cys-hTTR, m/z 13880.1 ± 2.8 Da, Δmass +118 Da), and S-glutathionylated hTTR (GSH-hTTR, m/z 14066.7 ± 2.2 Da, Δmass +305 Da). For the incubation with D-penicillamine an additional hTTR peak was detected and was assigned as hTTR-penicillamine adduct (m/z 13909.4 ± 0.6 Da, Δmass +148 Da). Likewise, the formation of a specific hTTR-MND adduct (hTTR-MND, m/z 13932.7 ± 3.0 Da, Δmass +171 Da) was recognized after the incubation of CL2008 transgenic worms with MND.

In native CL2008 transgenic worm samples (not stimulated with any substance) unmodified hTTR represented the predominant peak and only minor amounts of sulf-hTTR, cys-hTTR and GSH-hTTR could be detected (Figs 2, 3 and 4). However, the incubation of CL2008 transgenic worms with D-penicillamine and MND resulted in concentration-dependent changes in the PTPM pattern of hTTR. In detail, the incubation of CL2008 with 50 mM D-penicillamine resulted in the formation of significant amounts of hTTR-penicillamine adducts. Increasing amounts of D-penicillamine (100 mM) were further associated with an increase in the relative intensity of the respective hTTR adduct (Fig. 2D). The formation of hTTR-penicillamine adducts was accompanied by a relative increase of sulf-hTTR (Fig. 2B) and a decreased relative intensity of cys-hTTR (Fig. 2C). In contrast, the relative intensity of GSH-hTTR was not affected by D-penicillamine (data not shown).

With regard to MND, formation of significant amounts of hTTR-MND adducts was induced by MND concentrations as low as 200 μM. Furthermore, increased amounts of MND resulted in a concentration-dependent, consistent increase in the relative intensity of hTTR-MND adducts (Fig. 3C) that was accompanied by increased relative intensity of GSH-hTTR (Fig. 3B). Additionally, the relative intensity of cys-hTTR trended towards an increase, but these changes were not significant (data not shown). In contrast, sulf-hTTR was detectable only in minimal amounts in native CL2008 and CL2008 incubated with MND and was therefore not considered for data evaluation.

### Molecular modelling experiments

Given the identification of a specific hTTR-penicillamine adduct and a hTTR-MND adduct in the MALDI-TOF mass spectra, modelling experiments were performed to confirm the adduct formation. Specifically, a homology model of hTTR based on X-ray structures^10^ was used to study the putative reaction between the compounds D-penicillamine, MND and Cys10 from hTTR. We assumed that the reaction between D-penicillamine and the cysteine thiol group is an alkylthiol oxidation with disulfide as the final product. In contrast, MND and the cysteine thiol group most likely react *via* 1,4-Michael addition to a beta alkylether. Hence, these two types of chemical reactions were used in the covalent docking experiments and the formation of stable adducts could be confirmed.

**Figure 5:**
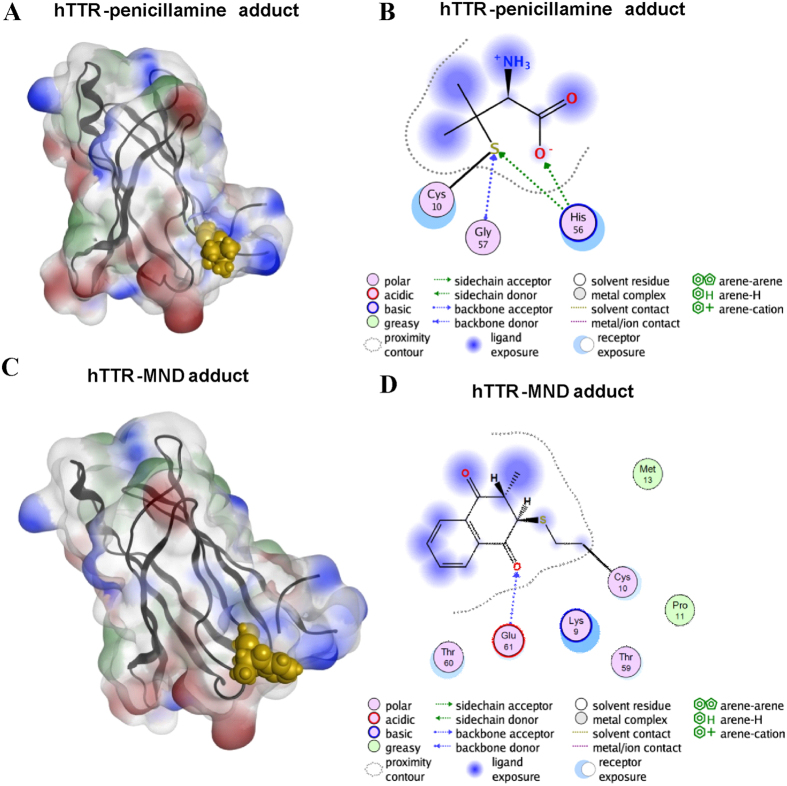
Molecular modelling experiments. Protein patch analysis surface models of the hTTR in complex with D-penicillamine (**A**) and MND (**C**). Polypeptide chain of hTTR is shown in black; ligands are shown in gold; positive, negative and hydrophobic areas are highlighted in blue, red and green respectively. The ligand models (**B** and **D**) show the covalent binding of D-penicillamine and MND with hTTR as well as interactions with other residues of the hTTR polypeptide chain at the end of the molecular dynamics experiments (30 ns).

In Molecular Operating Environment (MOE) the compounds (D-penicillamine, MND) were docked in a rigid receptor and also as inducted fit procedure. The two of them give plausible structures of covalent bonding in intermolecular complexes. The best-docked position was determined by comparing docking poses and considering the total energy value. Among several similar docking poses, the more energetically favorable conformation was selected (Fig. 5). The poses with the best energy value were examined by a molecular dynamics calculation (30 ns). Figure 5A shows the docking of D-penicillamine in the protein patch analysis surface model. The ligand model further indicated that the acidic residue Glu 61 interacts *via* hydrogen bond with the covalent bound D-penicillamine, and that Lys 9 as well as Thr 60 are involved in this process. After the molecular dynamics simulation, we noted a change in the interaction network; Glu 61 lost the interplay, while Gly 57 and His 56 became H-acceptors (Fig. 5B). The binding energy increases from −1.0 kcal/mol to −15.2 kcal/mol.

MND shows different orientation in the binding as demonstrated in the protein patch analysis surface model as well as in the ligand model (Fig. 5C,D). Glu 61 and Thr 59 are H-acceptor and H-donator with an energy value from −5.2 kcal/mol. After the 30 ns molecular dynamics simulation we lost energy to a value of −3.3 kcal/mol and only Glu 61 has a H-bond (Fig. 5D).

### N-acetylcysteine (NAC) partly reverts the toxic and oxidant effects of MND in CL2008

**Figure 6:**
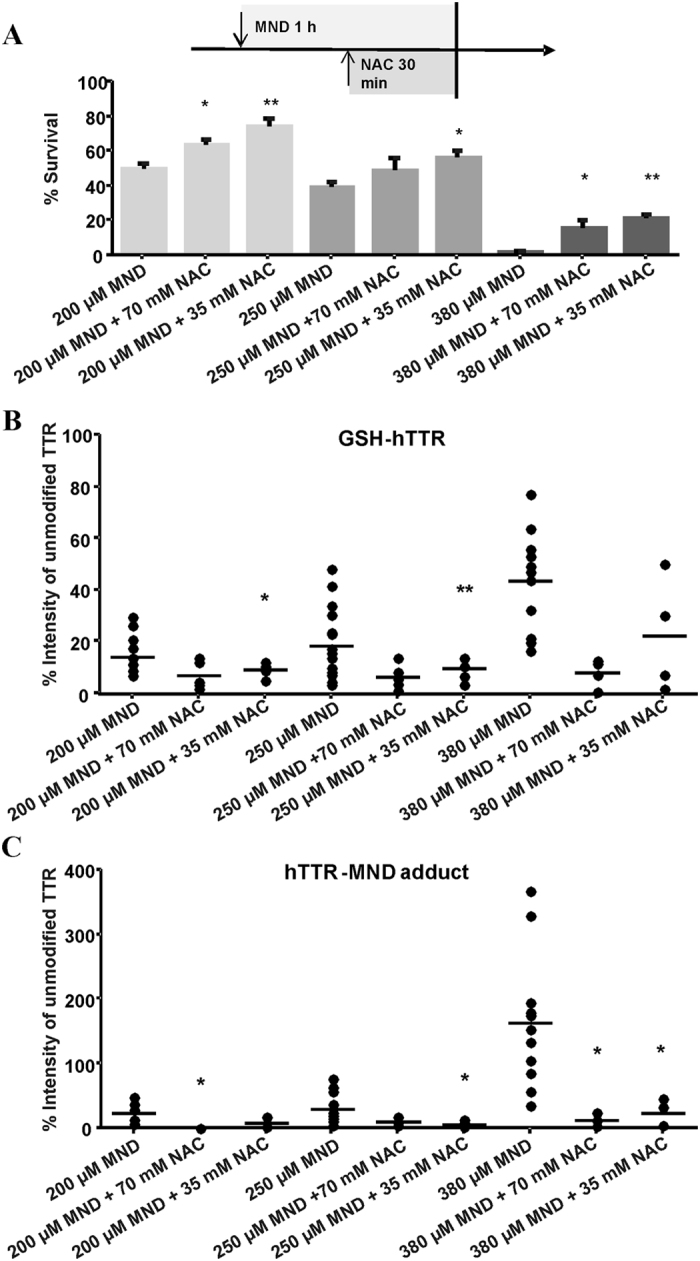
Co-incubation of CL2008 with MND and NAC after pre-treating with MND for 30 min. (**A**) Dose–response survival plot pre-incubating worms 30 min with MND following a 30 min co-incubation of NAC and MND. All values were compared to CL2008 worms incubated with the corresponding MND concentration only. Data are expressed as means + SD from at least four independent experiments. (**B,C**) Relative amounts (intensity of the unmodified hTTR [%]) of hTTR post-translationally modified at Cys10 in homogenates of L4 stage CL2008 worms pre-incubated 30 min with MND following a 30 min co-incubation of NAC and MND. Shown are mean values of at least four experiments each as an aligned dot blot (showing data points and the mean in form of a line). (**B**) Concentration-dependent effects of MND on the relative intensity of GSH-hTTR in CL2008. (**C**) Concentration-dependent effects of MND on the relative intensity of the hTTR-MND adduct in CL2008. **p < 0.01, *p < 0.05 versus CL2008 worms incubated with the corresponding MND concentration only.

The ability of the antioxidant NAC to revert the toxic and oxidant effects of MND was established by pre-incubating CL2008 transgenic worms 30 min with MND following a 30 min co-incubation with NAC and MND. As shown in Fig. 6A, co-incubating with NAC and MND resulted in an amelioration of the MND-induced lethality compared to incubation with MND alone.

Furthermore, MND-induced changes in the PTPM pattern of hTTR were reversed by NAC. With respect to GSH-hTTR, co-incubation with 35 mM NAC led to 5% and 9% reduction in comparison to 200 and 250 μM MND alone, respectively (Fig. 6B). In contrast, the co-incubation with 70 mM NAC and 200 μM and 250 μM MND, respectively, had no significant effects on the formation of GSH-hTTR adducts. Additionally, for the experiments with 380 μM MND neither incubation with 35 mM NAC nor with 70 mM NAC had significant effects on the relative amounts of GSH-hTTR.

With respect to the hTTR-MND adducts depicted in Fig. 6C, the effect of NAC was more obvious, resulting in 15%, 22% and 140% reduction in hTTR-MND adduct formation upon co-incubation with 35 mM NAC compared to the respective MND concentration alone. At 380 μM MND, the effect was most pronounced with the relative intensity of hTTR-MND adducts being substantially reduced by both 35 mM NAC and 70 mM NAC.

## Discussion

hTTR-related amyloidosis encompasses two forms of diseases including a familial and a sporadic amyloidosis. The familial arises from misfolding of a mutated hTTR (familial amyloid cardiomyopathy (FAC) or familial amyloidotic polyneuropathy (FAP)), whereas the sporadic, non-genetic disease is due to misaggregation of wildtype hTTR (senile systemic amyloidosis (SSA))^20^. Thereby, oxidative PTPMs at Cys10 have been shown to effectively destabilize the tetramer structure and facilitate wildtype amyloidogenesis, leading to the onset of SSA^16^. Consequently, it is imperative to monitor drug-mediated oxidative changes and the degree of oxidative PTPMs *in vivo*. Performing *in vivo* studies with *C. elegans* as a model system offers a unique approach to study drug-mediated oxidative PTPMs in a metabolizing system within the context of a whole organism. Furthermore, the genetically tractable nematode permits studies on drug-mediated effects on hTTR since the transgenic strain CL2008 is expressing high levels of hTTR, which is secreted from muscle cells and distributed throughout the animal^29^. In wildtype worms there are protein sequences listed as TTR-like in the databases, with a particularly large number of 59 genes^30^. While most are uncharacterized with less information known about their function, for example “TTR-1” has been shown to influence aging in *C. elegans*, whereas “TTR-52” has been implicated for efficient cell corpse engulfment^31^. However, the TTR-like proteins occupy a relatively low sequence identity compared to hTTR^32^ and have not been detected by real time RT-PCR and immuno blot analysis within this study. Due to the high levels of hTTR in relation to the total protein content, the number of requisite worms to study oxidative PTPMs of hTTR could be reduced, enabling in combination with the worms quick life cycle to carry out high-throughput analyses. Additionally, studies can be performed in L1 larvae as well as L4 larvae showing indistinguishable hTTR expression normalized relative to afd-1/β-actin mRNA. Thus, this model system offers an opportunity to study possible developmental effects of drug-mediated oxidative PTPMs of hTTR.

In this context, the MALDI-TOF-MS analysis of hTTR isolated from CL2008 transgenic nematodes revealed that under native conditions hTTR is mainly present in its unmodified form. This is in contrast to hTTR obtained from human serum that usually presents a distinct PTPM pattern mainly characterized by adducts with cysteine, cysteine-glycine and GSH^10^,^15^. Thereby, the formation of these mixed disulfides is most likely promoted by the high availability of the respective aminothiols in human metabolism and circulation^33^. With respect to *C. elegans,* no systematic analysis of the aminothiol content has been carried out to date. However, it is reasonable to assume that the total amount of aminothiols is rather low. Hence, the predominance of unmodified hTTR in CL2008 transgenic nematodes is most likely a consequence of missing reaction partners and is likely not caused by differences in hTTR reactivity.

Furthermore, the suitability of the model system to study drug-mediated oxidative PTPMs of hTTR was verified by exposing CL2008 transgenic nematodes to D-penicillamine or MND. Introduced first in 1956, D-penicillamine is a thiol-containing drug classically used in the treatment of Wilson’s disease, rheumatoid arthritis, and cystinuria^34^. With respect to its reactivity, D-penicillamine has been invoked as potential protective agent against protein carbonyl formation due to its irreversible reactivity towards the electrophilic α-dicarbonyls^35^. In the present study we were able to demonstrate for the first time that challenging *C. elegans* CL2008 transgenic worms with D-penicillamine results in a concentration-dependent formation of stable adducts with hTTR. Thereby, the binding is most likely attributed to the formation of a covalent disulfide bond *via* the thiol groups present in both compounds, which has been shown by molecular modelling experiments. Interestingly, the formation of hTTR-penicillamine adducts in CL2008 nematodes was accompanied by changes in the overall PTPM pattern of hTTR with a decrease of cys-hTTR and an increase of sulf-hTTR. The underlying mechanisms for these modulations remain to be elucidated. Nevertheless, taking into account that all quantifications were performed in relation to unmodified hTTR, the increase in sulf-hTTR is most likely related to the consumption of unmodified hTTR for the formation of hTTR-penicillamine adducts. In contrast, for cys-hTTR, it seems reasonable that the changes are attributed to a thiol-exchange reaction induced by the addition of D-penicillamine.

The oxidant MND (vitamin K3; 2-methyl-1,4-naphthoquinone) is a synthetic derivative of vitamin K1 which displays antitumor activity against a variety of tumor cells and is known as a strong oxidant particularly targeting mitochondrial structures^36^. Two processes might be invoked in the reduction of MND; one-electron reduction or two-electron reduction. Firstly, MND is metabolized by cytochrome P450 reductase resulting in the formation of a semiquinone radical, which can be oxidized back to the quinone in the presence of molecular oxygen. This redox cycle leads to the formation of superoxide radicals inducing a variety of effects, including depletion of GSH, induction of single-stranded DNA breaks and apoptosis^37^,^38^. Secondly, stable and nontoxic hydroquinones are generated in a two-electron reduction by DT-diaphorase and carbonyl reductase of MND leading to its detoxification^39^. Furthermore, the addition chemistry of quinones involves redox transitions that yield a reduced quinone conjugated to nucleophiles, including thiol groups in glutathionyl-hydroquinone conjugates^40^. The conjugation to nucleophiles was also corroborated in this study, as we identifed a MND-hTTR conjugate by MALDI-TOF-MS. Thereby, the formation of the MND-hTTR adducts was concentration-dependent. The underlying mechanism predicted by the modelling experiments is consistent with covalent binding due to a 1,4-Michael addition. The formation of MND-hTTR adducts is likely reflective of the interaction with lysine or cysteine residues. However, due to the higher electron density of sulfur atoms, the conjugation *via* cysteine residues is more likely. Furthermore, only mono-MND-hTTR adducts were detected by MALDI-TOF-MS, and considering that hTTR reveals only one cysteine residue but eight lysine residues, further corroborating the hypothesis that the covalent binding of MND is based on cysteine interaction^41^. The modelling experiments further suggest that the MND bounding of hTTR might result in a destabilization of the protein’s conformation which has to be proven in further modelling experiments as well as in *in vivo* studies.

Nevertheless, in contrast to D-penicillamine, incubation with MND was associated with a substantial increase in the relative amount of GSH-hTTR while other hTTR variants remained unchanged. The increase in GSH-hTTR levels might be attributed to the decrease in unmodified hTTR due to the formation of MND-hTTR adducts. Nevertheless, due to the pro-oxidant nature of MND, the increase in GSH-hTTR might also be a consequence of increased GSH formation and it remains to be verified in future research. This hypothesis is corroborated by studies reporting that protein glutathionylation could be observed in response to oxidative stress which can further affect both the stability and activity of target proteins^42^,^43^.

NAC is a mild and effective antioxidant that promotes GSH synthesis, but non-antioxidant mechanisms have also been suggested. In the present study, we were able to demonstrate that NAC, at least in part, counteracts MND-induced toxicity as indicated by improved survival of CL2008 transgenic nematodes. Additionally, MND-induced changes in the hTTR PTPM pattern were mostly reversed by NAC. These findings are consistent with previous studies in which NAC supplementation of *C. elegans* increased the resistance to environmental stressors^44^,^45^. Additionally, cell culture based studies have shown that the antioxidant NAC effectively protected against MND-induced oxidative stress^46^,^47^. The detoxifying action of NAC observed in the present study might be related to its property as GSH precursor^48^. However, the reduction of GSH-hTTR contradicts this assumption. Therefore, it might be due to a chemical detoxification since 1,4-naphthoquinones as MND are known for their reactivity towards the thiol group of NAC^49^. NAC covalently binds to 1,4-naphthoquinone derivates reducing the abundance of free thiol groups which is reflected by decreased binding of MND to hTTR in the presence of NAC. Nonetheless, further studies are necessary to decipher the underlying mechanism.

## Conclusion

Altogether, this is to our best knowledge the first study with *C. elegans* as model system to study kind and degree of drug-mediated oxidative PTPMs of hTTR by immunoprecipitation and MALDI-TOF-MS analysis. Incubation of worms expressing hTTR with D-penicillamine and MND offers considerable insight into the role of specific cysteine-modifying oxidative agents in disease etiology. Using *C. elegans* allows for high-throughput analyses in a whole *in vivo* organism with conserved pathways of oxidative stress and oxidative stress response. Due to its genetic manipulability a tremendous advantage is further offered by studying hTTR in all larval stages. Thus, our studies offer insight into the prediction of drug-mediated oxidative PTPMs of hTTR, which is imperative since oxidation can influence hTTR functions in several diseases including SSA. Findings in *C. elegans* hold future promise of delineating the genetic and molecular mechanisms involved in disease progression due to hTTR misfolding, which will be helpful in deciphering counteracting therapy strategies.

## Methods

### *C. elegans* strain and handling

The N2 Bristol strain was provided by the Caenorhabditis Genetics Center (CGC; University of Minnesota). The construction of the transgenic nematode strain CL2008, which expresses high levels of hTTR, has been previously described^29^. The *C. elegans* strains were propagated at 20 °C on Nematode Growth Medium (NGM) plates or 8 P plates seeded with either *Escherichia coli* strain OP50 or NA22, respectively. Synchronous L1 populations were obtained by treating worms with an alkaline bleach solution (1% NaClO and 0.25 M NaOH)^45^. L1 larvae were placed on OP50-seeded NGM plates after hatching and experiments were performed using L4 stage nematodes unless otherwise noted.

### Relative hTTR gene expression in CL2008

Real time RT-PCR was performed for quantification of the hTTR mRNA level in eggs, L1 stage and L4 stage CL2008 larvae. Total RNA was isolated using the Trizol method as described before^50^. Briefly 1 mL Trizol (Life Technologies) was added to each tube containing about 60 000 eggs, 20 000 L1 stage or 4 000 L4 stage worms, followed by three cycles of freezing in liquid nitrogen and thawing. The protein and other impurities were separated from the nucleic acids by adding 200 μL chloroform. Afterwards the precipitate obtained by using isopropanol and glycogen (Life Technologies) was washed with 75% ethanol. 1 mg isolated RNA was used for cDNA synthesis applying the High Capacity cDNA Reverse Transcription Kit (Life Technologies), as per manufacturer’s instructions. Real time RT-PCR was conducted in triplicate wells using TaqMan Gene Expression Assay probes (Life Technologies) for afd-1 (assay ID: Ce02414573_m1) (actin homolog) and hTTR (assay ID: Hs00174914_m1). The relative gene expression was calculated with afd-1 as housekeeping gene for normalization after determining the fold difference using the comparative 2 ^−ΔΔCt^ method^51^.

### Determination of total worm protein and relative hTTR protein level in CL2008

4 000 L4 stage worms were pelleted by centrifugation at 1 600 rpm for 2 min and washed three times in M9 buffer containing 0.01% Tween. Afterwards, the worm pellet was re-suspended on ice in 70 μL PBS and temporarily frozen in liquid nitrogen. The extract was homogenized by sonication and centrifugation and the protein content was determined according to Bradford^52^. The samples were diluted with sample buffer and used to quantify protein levels of hTTR and actin as control (mouse-anti actin antibody (Abcam), 1:400) by SDS-PAGE with subsequent immunoblot analysis as previously described^53^,^54^.

### Preparation of standard solutions

A MND stock solution was prepared in DMSO (Sigma-Aldrich, Taufkirchen, Germany). D-penicillamine (Sigma-Aldrich) and NAC (Sigma-Aldrich) were dissolved in M9 buffer (KH_2_PO_4_; Na_2_HPO_4_; and NaCl). To prevent oxidation, fresh stock solutions were prepared shortly before each experiment.

### Treatment, dose-response curves and extraction of hTTR from worm homogenates

Treatment was performed using 4 000 L4 stage worms. The L4 stage nematodes were exposed to MND, D-penicillamine or NAC in siliconized tubes for 1 h in M9 buffer containing 0.01% Tween. Worms were then pelleted by centrifugation at 1 600 rpm for 2 min and washed three times in M9 buffer containing 0.01% Tween. The lethal dose 50% (LD_50_) was determined by transferring and pre-counting 30–50 worms to OP50-seeded NGM plates in triplicate. 24 h post-treatment, the total number of surviving worms was scored as a percentage of the original plated worm count. After seeding the aliquot worms for the lethality assay the remaining worms were pelleted, re-suspended on ice in 70 μL PBS and temporarily frozen in liquid nitrogen. Finally, the extract was homogenized by sonication and centrifugation. The extracted worm homogenates could be stored at −80 °C until immunoprecipitation and MALDI-TOF-MS analysis of hTTR.

### Immunoprecipitation and MALDI-TOF-MS analysis of hTTR

Immunoprecipitation of hTTR from CL2008 homogenates, MALDI-TOF-MS analysis and mass spectra processing were performed as previously described^10^,^15^. All spectra were screened for the presence of unmodified hTTR and the hTTR variants sulf-hTTR, cys-hTTR, GSH-hTTR as well as hTTR-MND adducts and hTTR-penicillamine adducts, as shown in Fig. 4. For semi-quantitative analysis, the peak intensities of the hTTR variants were expressed as percentage of unmodified hTTR.

### Molecular modelling experiments

Molecular docking and energy minimization experiments were performed using the MOE molecular modelling program 2015.10^55^ and Yasara 15.11.18^56^,^57^. The homology was built in the same procedure as used before^10^. Chemical structures were created in ChemBioDraw Ultra14.0 (Perkin Elmer, Waltham, MA) and transferred to the MOE database. Ligands were then energy-minimized using the MMFF94 force field option with the restriction to preserve original chirality of the molecules and a root mean square deviation (RMSD) of 0.01 kcal/mol Å^58^. The LowmodeMethod was used for conformation search in standard configuration (only D-penicillamine). Molecular docking examination was done with a molecular dynamics simulation in Yasara. The stereochemistry quality aspects of the resulting models were checked *via* the MOE program 2015.10. RMSD from starting to end conformation in the molecular dynamics calculation was estimated with MOE. The trajectory in the molecular dynamics calculation was analyzed with Yasara (modified scripts).

The MOE docking protocols for rigid receptor and inducted fits were used in this study. The parameters for covalent docking were as follows: Experiment 1: reactant – D-penicillamine, functional group – alkylthiol, class – oxidation, product – disulfide; Experiment 2: reactant – MND, functional group – Michael acceptor, class – 1,4 addition, product – beta alkylether.

Rigid receptor or inducted fit site and reactive site selected thiol refinement: GBVI/WSA dG scoring function^59^.

The optimal complex for each ligand and receptor was then subjected to MD using Yasara dynamics amber03 force field^60^. A simulation cell was constructed around the hTTR covalent-bounded model (2*7.5 Å larger than the model) with a 7.9 Å real space cut-off for the electrostatic force calculated *via* the Particle Mesh Ewald method. The pKa values of the ionisable groups were predicted and assigned protonation states based on pH 7.4 (temperature = 298 K, density = 0.997). The cell was filled with water and the amber03 electrostatic potential was estimated at all water molecules, those with the lowest or highest potential was turned into sodium or chloride counter ions till the cell was neutral. A short steepest descent minimization was done to remove severe bumps followed by simulated annealing minimizations at 298 K. Molecular dynamics simulations were done with amber03 force field at 298 K and 0.9% NaCl in the simulation cell for 500 ps to refine the models. For further analysis simulation snapshots were captured every 25 ps over the simulation time from 30 ns.

### Statistical analysis

Dose–response lethality curves and all aligned dot blots (showing data points and the mean in form of a line) were generated using GraphPad Prism (GraphPad Software Inc.). Thereby, in case of MND exposure a sigmoidal dose–response model with a top constraint at 100% was used to draw the lethality curves and determine the respective LD_50_ values (values represent the respective MND dosing that induce 50% reduction in survival). In order to compare the results regarding the concentration dependent effects of the tested drugs (D-penicillamine, MND or co-incubating NAC and MND) on the PTPM patterns of hTTR in CL2008 a Kruskal–Wallis test using a Dunn’s multiple comparison post-hoc test was conducted.

## Additional Information

**How to cite this article**: Henze, A. *et al.* *Caenorhabditis elegans* as a model system to study post-translational modifications of human transthyretin. *Sci. Rep.* **6**, 37346; doi: 10.1038/srep37346 (2016).

**Publisher’s note:** Springer Nature remains neutral with regard to jurisdictional claims in published maps and institutional affiliations.
